# Integrating Environmental Context into DHS Analysis While Protecting Participant Confidentiality: A New Remote Sensing Method

**DOI:** 10.1111/padr.12222

**Published:** 2018-12-19

**Authors:** Kathryn Grace, Nicholas N. Nagle, Clara R. Burgert‐Brucker, Shelby Rutzick, David C. Van Riper, Trinadh Dontamsetti, Trevor Croft

## Introduction

Understanding the ways that people live given certain environmental conditions is of central concern to researchers in health, development, population, climate change, and other related fields (see Grace et al. 2014; Balk et al. 2005; de Sherbinin 2011). One major source of data on health and development is the USAID‐funded Demographic and Health Surveys (DHS) program. DHS is a major source of population and health data for the poorest countries in the world and provides high‐quality and detailed data on individual health outcomes—particularly outcomes related to maternal and child health. The primary sampling unit in the DHS are villages or village “clusters.” Cluster size can vary but contains a number of households within a geographic area who participated in the survey. Since many of the data included in DHS are personal and potentially sensitive, the DHS maintains confidentiality of the respondents by shifting the spatial coordinates of the cluster in the published data (Burgert et al. 2013). The spatial coordinates for rural locations are displaced by 0–5 km in any direction. Additionally, a small fraction of coordinates, 1 percent, are randomly shifted up to 10 km. For urban locations, the displacement is up to 2 km only. DHS recommends that researchers average any environmental data over a 5–10 km buffer around each DHS rural cluster with the specific community falling somewhere within the disc around each point (Perez‐Heydrich et al. 2016). This approach to maintaining confidentiality while collecting survey information has been adopted by other international organizations as well (e.g., World Bank's Living Standards Measurement Study).

Building on the rapid growth of literature around activity space, the geographic theory of close things being more alike (Tobler's First Law), as well as the understanding that people interact disproportionately with the landscape immediately surrounding a settlement, we propose an alternative method for evaluating environmental and contextual variables (Tobler 1970; Miller, 2004). Instead of calculating a 5–10 km buffer around each published point, we propose that the user selects a settlement near the DHS’ published cluster location and measures the environmental conditions around the settlement using a buffer much smaller than 10 km. We assume that the “true” context is a small, precise buffer around the correct settlement. We hypothesize that a small, precise buffer around an incorrect settlement is a better measure of truth than is an overly large buffer around the published point. Settlements can be identified through interpreting remotely sensed imagery. Corresponding features—for example, types of land‐use strategies or adjacency to reservoirs for irrigation—can be more easily identified and evaluated when using a much more precise buffer. While the settlement that is being used to provide this contextual information is likely not the original DHS cluster, it is a neighbor of the cluster and we assume that neighboring settlements are more similar to each other than to the broader environment in which they are situated. We theorize that this approach will introduce less measurement error than the larger 10 km buffer.

To test this theory, we select three countries that are topographically diverse and that represent unique regions of the world—Burkina Faso, Kenya, and Tajikistan. As with most of the poorest countries in the world, these countries are heavily dependent on the landscape to produce food and earn money. However, each of these countries is quite distinct from the others in terms of environmental characteristics (rainfall and topography) and cultural characteristics (the types of crops produced as well as the farming strategies used to produce the crops). We select these countries to develop a thorough understanding of how our methodology will function under different settings. We evaluate a remotely‐sensed estimate of cultivated area and vegetation features of the DHS clusters using the 5–10 km buffer approach and our proposed neighboring settlement approach. We compare these to the true values through the use of the actual, confidential, locations of settlements in the DHS sample.

## Background

### Scale in Geography

Scale in geography refers to the spatial size of objects or processes. Geographers often find it helpful to distinguish between cartographic scale, analysis scale, and phenomenon scale (Montello 2001). Cartographic scale is not immediately relevant to this study. Analysis scale is the scale at which data are collected or used. Sometimes, the analysis scale is not controlled by the researcher, such as when economic statistics are produced nationally, or when the resolution of satellite imagery is fixed by the sensor. At other times, however, the scale is controllable by the researcher, such as when the DHS recommends that a 10 km buffer be placed around public geocodes. The only justification for choosing a 10 km buffer is that presumably it includes the true point and overlaps significantly with the 10 km buffer around the true point. But other buffer sizes could be chosen. Phenomenon scale refers to the size at which geographic structures or processes exist. For example, in the case of individuals in the DHS sample, their daily lives occur within a particular geographic scale. The size of the local environment that produces crops for food and cash for a village has a particular scale. The recent explosion of thinking and research on activity spaces is fundamentally about phenomenon scales (see Perchoux et al. 2013 and Zenk et al. 2011 and many others) and has been explored in less developed countries most often in ways that relate to geographic access to services (for some examples see Yao et al. 2013; Buor 2003; Tanser et al. 2006).

### Geographic Scale Matters

We imagine sparsely settled rural environments in which the natural landscape is “lumpy.” That is, there are places which constitute the immediate environmental context of a village, and then there are the in‐between places that do not strongly constitute the context of any village. If this is so, then it is reasonable to assume that the “lumps” formed by settlements and their environs are more similar to each other than they are to the in‐between places. Survey theory tacitly makes this assumption when it identifies villages as the primary sample unit; i.e., that villages are statistically exchangeable for one another. An alternative survey frame, which maintains that space is homogeneous, would just pick random spatial coordinates uniformly. But we know that geographic space is lumpy, and this motivates us to hypothesize that a buffer around a settlement, any settlement, is a better measure of local context than a buffer around a non‐settlement.

**Figure 1:**
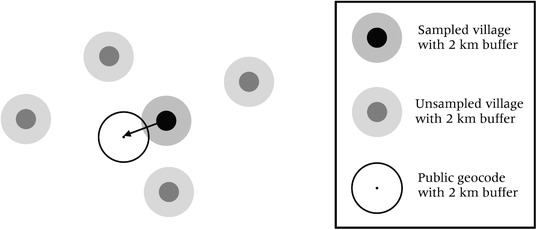
Schematic of sampled and unsampled villages buffered by 2 km, and a public geocode point buffered by 2 km

**Figure 2:**
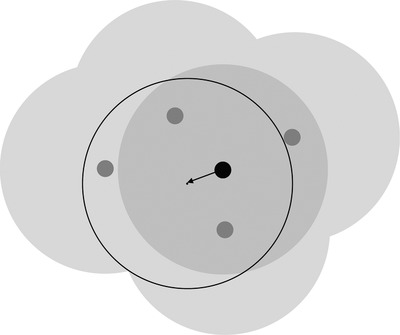
The same villages as in Figure 1, except buffered by 10 km NOTE: If 10 km is the appropriate scale, then the difference between large buffers is not as great as the difference between small buffers.

This, of course, depends on both the scale of processes in rural settlements and on the degree of lumpiness in the natural environment. When the buffers are small, we expect that there may be a large difference between the local context of settled and non‐settled places (Figure 1). In contrast, as the buffers get larger, the difference between the context of settled and non‐settled places becomes less apparent (Figure 2). Similarly, in places with relatively even rural development or cultivation, we expect to see less difference between the local context of settled and non‐settled places.

When using spatial population data like the DHS, these issues of scale obviously vary according to the level of development, infrastructure, and resources, and can have important implications on research design and analysis. For example, in a developing country where walking is the main mode of transit, intensive cultivation is much more likely within a 2 km radius than a 10 km radius. If so, then we suggest that a 2 km buffer is a more appropriate measure of the relevant environmental context of a settlement than a 10 km buffer. In this study, we will not address the “correct” geographic scale, but we will explore whether the analysis scale matters (it does) and suggest an alternative approach for measuring environmental context.

### Applications

Researchers in physical, social, and health sciences have increasingly focused on merging data, methods and theory from across multiple disciplines. For those interested in any aspect of human‐environment interaction, merging large‐scale survey data (e.g., Demographic and Health Surveys (DHS) data or World Bank Living Standards Measurement Study (LSMS)) with climate data or landscape data requires problem solving. One of the ongoing challenges relates to understanding the consequences of the displacement processes used with spatially‐referenced survey data. Strategies like those explored here will provide some possible suggestions into how remotely‐sensed data can be used with survey data to better contextualize and differentiate the communities where people live. This approach can help researchers explicitly incorporate context, culture, and spatial thinking into their analyses while guarding the confidentiality of the respondents (VanWey et al. 2005). While researchers are empirically and conceptually exploring strategies to merge survey data and physical/climate data to investigate a variety of outcomes (see, for example Noor et al. 2009; Tatem et al. 2007; Tatem et al. 2012; Tanner et al. 2015; Nawrotzki and Bakhtsiyarava 2017; Brown et al. 2014; Shively et al. 2015), most research does not explore strategies for incorporating the spatial displacement (see Dorélien et al. 2013 for a discussion of related urban DHS issues). We aim to produce an alternative approach that accounts for the displacement of geocodes.

## Data

### DHS data

Our analysis is organized around the most recent spatially referenced DHS data for each of the three countries under study. In each country, we have restricted the sample to rural settlements since our interest here is on (natural) environmental context. Public DHS records contain a geocoded coordinate for each sampled settlement cluster; however, these coordinates are displaced up to 10 km prior to publication and do not identify an actual settlement. For our analyses, we have access to the true settlement location. We will calculate the vegetation measures (in this case remotely sensed based estimates of vegetation) for each of the “true” DHS spatial coordinates as well as the displaced (publicly available) coordinates.

### Settlement Locations

We use Digital Globe^1^ (∼35 cm resolution) remotely sensed imagery to identify the location of settlements near the publicly‐released DHS cluster geocodes. This highly detailed imagery reveals landscape characteristics—namely clusters of dwellings, agricultural plots, and road networks—that signify the presence of human settlement. Recent imagery with low cloud cover, so that villages can be identified clearly, is used in the settlement identification. Specific details on the strategy will be described in the methods section below.

### Normalized Difference Vegetation Index (NDVI)

We use Landsat 8 Normalized Difference Vegetation Index (NDVI) (USGS 2017) to estimate community‐level food production for each spatially referenced DHS cluster. The Landsat 8 satellite was launched in 2013 and images the Earth's surface every 16 days at 30 m resolution. The range of NDVI is –1 to 1, where a value of 1 indicates more greenness (or vegetation). NDVI is a commonly used measure within the applied remote sensing and food security communities and by remote monitoring systems to measure the amount of vegetation growth, agricultural production, or to estimate food production (see Tucker 1979; Husak et al. 2008; Grace et al. 2017; Grace et al. 2014; Brown et al. 2014). It is an ideal variable to test our theory as agriculture reflects landscape characteristics and human response to, and engagement with, the environment. For the countries under study (like most developing countries), no other annual crop data exist, including annual maps of cropped area. NDVI provides just one of multiple variables that could be investigated in this way. The most important consideration is that the variable under study reflects the general way that people who are near to each other would engage with the environment. The spatial scale must also be relatively fine so that actual spatial variation within a specified buffer is present.

### Famine Early Warning System (FEWS NET) Livelihood Zones

We also use the Famine Early Warning System Network's (FEWS NET)^2^ livelihood zones data. These maps and associated reports are developed based on quantitative and qualitative information related to land use, food production, climate, economics, trade routes, and historical information. They provide insight into the strategies that people within a particular area generally use to procure food or income and are useful for contextualizing the role of rainfall in agricultural production. The maps and reports are freely available and have been used in a wide range of research (see Brown et al. 2014; Grace et al. 2014; Shively et al. 2015; Grace 2017).

## Methods

Our primary goal is to determine correct ways to measure the environmental context of rural settlements in the Demographic and Health Surveys while also preserving the confidentiality of the individuals and communities selected. In this research, we are interested in the integration of DHS data with satellite imagery to determine the local environmental context of a community. We restrict our study to the most recent surveys from Burkina Faso (2010), Kenya (2014), and Tajikistan (2012) (ICF 2010; 2012; 2014). These countries were selected based on their representation of a diverse range of livelihood strategies (see FEWS NET livelihood zones), land use patterns, and quality (or lack thereof) of available geospatial census data.

### Settlement Sample Selection

**Figure 3:**
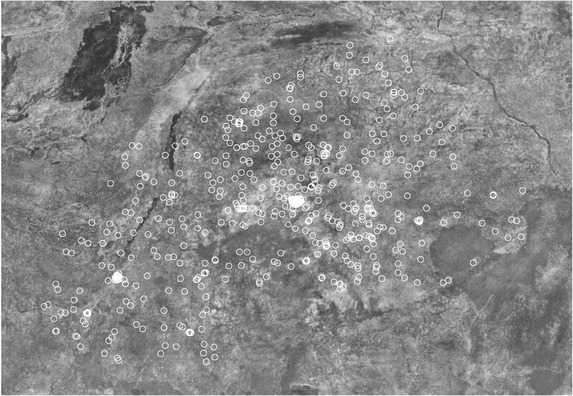
5 km buffers around Burkina Faso 2010 DHS clusters

In each country, we identify settlements around DHS public geocodes using high resolution (∼35cm) satellite imagery from Digital Globe taken within (approximately) 1–3 years of the survey. We use the DHS rural/urban coding to select out rural clusters. We then generate a random sample of DHS clusters from each country, draw a 5 km buffer around the public geocode point, and overlay the buffer on the Digital Globe imagery. Figure 3 displays the high‐resolution imagery overlaid with rural DHS cluster buffers for Burkina Faso.

**Figure 4:**
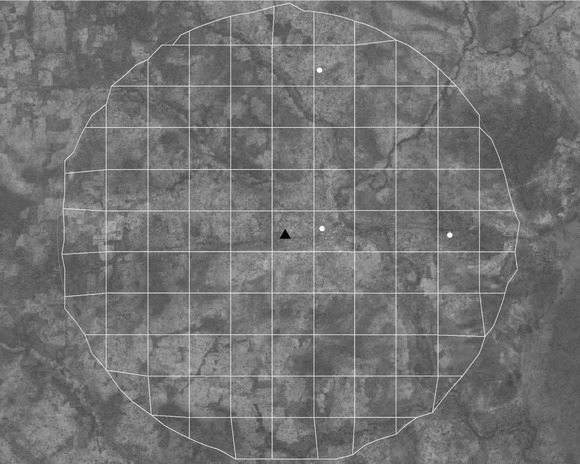
Fishnet over DHS public geocode (triangle) and identified settlements (circles)—Burkina Faso

**Figure 5:**
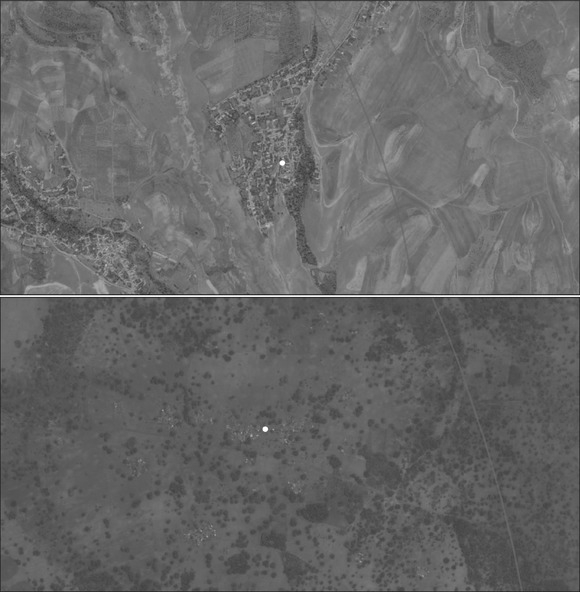
Identified settlement within 5km of a DHS survey location —Tajikistan (top), Burkina Faso (bottom)

We then create a “fishnet” within each buffer to facilitate identification of the settlements. Multiple settlements could be identified within a buffer and we aimed for at least one settlement identification per buffer. In Figure 4, the fishnet, the DHS cluster, and the identified settlements are shown. Note that at this scale, the settlements are nearly impossible to identify and require the analyst to zoom in for a closer look (Figure 5).

Culture, topography, politics, level of development, community infrastructure, and livelihood/economic systems may influence the ways that settlements form and households cluster. We looked for specific features—road/path networks and housing structures that seemed comparatively dense/populated enough to justify a settlement cluster. Figure 5 provides a zoomed in view of an identified settlement in Burkina Faso and in Tajikistan.

### Generation of alternative spatial coordinates

Our prime hypothesis is that it is more accurate to measure spatial context as centered around actual settlements (even the wrong settlements) than it is to measure it as centered on a random (non‐settlement) location. To make this comparison, we identify three possible locations for the rural cluster: the true location^3^ (T), the current location (0) that is generated by randomly displacing the true location by 0–10 km, and the alternative location (1) that is the set of settlements within 5km of the current location, as identified by manual interpretation. To reduce interpreter burden, up to three locations were identified.

### Calculating environmental context

From the Landsat 8 satellite imagery, we calculated the median NDVI around these locations; at buffer sizes of 2 km, 5 km (to capture the activity space for the settlement and true locations), and 10 km (for the current location). Ten km is selected because that is the current recommendation given by DHS. Two and five km are selected because they more closely represent the activity space of rural residents in these countries; however, this distance is not tested or calibrated here.

Landsat 8 images a scene every 16 days. For each DHS cluster, we scanned the Landsat 8 archive to find all scenes acquired within August, September, or October, and with less than 5 percent cloud cover. In some instances, no image could be found, and we eliminated that point. When more than one image could be found, we selected the image with the lowest cloud cover. Apart from this, we did not filter out cloudy pixels from the scene. To moderate the effect of clouds, we used the median, rather than mean, to characterize NDVI around a settlement. For the settlement sizes considered here, we do not believe that there will be any systematic bias between the cloudiness of settled and non‐settled pixels (note that with remotely sensed imagery, smoke from cooking fires, for example, would not create the type of cloudiness that would exclude a pixel (Gao et al. 2006)).

We did not search for images to correspond with the time of the DHS survey. Since this is a methodological study meant to look at data approaches, and we are not using the DHS survey information, this is not a problem. We are interested only in the question of whether the environmental context of a settlement in the DHS sample is different than the context around the public geocodes, and for this question it suffices to simply choose images around these points in any year. Problems may arise due to the gap between the Landsat 8 imagery and the Digital Globe imagery used to identify settlements if settlements are constructed or vacated during this gap^4^; however, the chances of this occurring in two or three years is so low as to be ignorable.

### Hypothesis Testing

In application, we aim to detect any difference between the context around the public point and the true point. Given the importance of scale and context, specifically, we compare 5 and 10 km buffers of the public point with the 2 and 5 km buffers of the true point. We aim to determine if the alternative solution we proposed is either statistically different from the value of the true location or from the DHS recommendation. In other words, does our suggested alternative improve upon the DHS recommendation? Five and ten km buffers are selected as they reflect the DHS recommendations (Perez‐Heydrich et al. 2016).

We test the following two hypotheses:

1. A 5 km buffer around the alternative (1) is closer to the true 5 km buffer (T) than is the DHS recommended 10 km buffer around public point (0)
  H0: MSE x15−xT5= MSE x010−xT5 vs  HA : MSE x15−xT5< MSE x010−xT5
2. A 2 km buffer around the alternative (1) is closer to the true 2 km buffer (T) than is the finer DHS‐recommended 5 km buffer around public point (0)
  H0: MSE x12−xT2= MSE x05−xT2 vs  HA : MSE x12−xT2< MSE x05−xT2

where *x* indicates the vegetation estimate, the subscript indicates the spatial location of the buffer center, and the superscript indicates the radius of the buffer. As a reminder, *T* indicates the true location, 0 is used to indicate the publicly‐available DHS geocodes, and 1 refers to our alternative settlements approach.

In other words, we are comparing the differences in the vegetation values calculated for the settlements and the true locations to the differences calculated between the DHS public geocodes and the true locations. The null hypothesis states that these differences would be statistically equivalent, while the alternative hypothesis states that the difference between the settlement location and the true location would be statistically smaller than the difference between the public geocode and the true location. The difference between the two hypotheses is the buffer size. In hypothesis 1, we are using the larger buffer size recommended by DHS (10 km) and comparing it to a 5 km activity space. In hypothesis 2, we are using the smaller buffer size recommended by DHS (5 km) and comparing it to a 2 km activity space. This approach is consistent with our interest in determining if a smaller buffer around an incorrect settlement is a better estimate than the larger “catch‐all” buffer.

We use permutation tests to compare the error between the two methods. Compared to a t‐test or F‐test, a permutation makes fewer assumptions about the distribution and allows more flexibility in the choice of a test statistic. As a test statistic, we choose the median of error ratios: R = median($|x_{1}^{5}-x_{T}^{5}\left|/\right|x_{0}^{10}-x_{T}^{5}|$), using hypothesis 1 as an example. We choose the median because it is robust to the presence of small numbers in the denominator. The null hypothesis of no difference between methods is equivalent to the hypothesis that R = 1. To evaluate the significance of our sample ratio R, we permute the labels of the *X*_1_ and *X*_0_ within each cluster (i.e., we preserve the pairing within clusters). Under the null hypothesis that the errors are equal, these labels are not meaningful, and they are therefore randomly assigned. We repeated this permutation 10,000 times, calculating the test statistic, R, under each permutation. Then, using the distribution of these permuted R statistics, we calculated the tail probability of the sample R value.

## Results

### Burkina Faso

For the first hypothesis, when comparing differences irrespective of livelihood zone, we reject the null hypothesis (p<0.01). This finding suggests that the difference in the vegetation values, when comparing the settlements and true locations, is statistically smaller than the difference between the DHS recommended approach and the true locations. The second hypothesis, which compares estimates using smaller buffers to capture activity space, produced similar statistically significant findings (p = 0.03), again suggesting that the settlement approach produces less of a difference when compared to the true locations versus the DHS‐recommended approach. Table 1 provides the mean values, standard deviations, and sample sizes across the country and according to each livelihood zone.

**Table 1:** Means and standard deviations (in parentheses) of median NDVI calculated for the settlement location, for the true location, and for the public DHS cluster location—Burkina Faso

	Settlements		Public Points		True Points		
Livelihood Zone	2 km	5 km	5 km	10 km	2 km	5 km	N
South tubers and cereals (Z1)	0.263 (0.034)	0.269 (0.032)	0.267 (0.032)	0.270 (0.030)	0.269 (0.040)	0.271 (0.035)	11
Southwest fruits, cotton, and cereals (Z2)	0.301 (0.046)	0.305 (0.034)	0.304 (0.037)	0.307 (0.031)	0.297 (0.043)	0.303 (0.036)	19
West cotton and cereals (Z3)	0.241 (0.018)	0.249 (0.014)	0.246 (0.015)	0.252 (0.018)	0.241 (0.016)	0.248 (0.013)	14
West cereals and remittances (Z4)	0.232 (0.018)	0.239 (0.015)	0.238 (0.015)	0.238 (0.014)	0.233 (0.021)	0.238 (0.016)	11
Central plateau cereals and market gardening (Z5)	0.197 (0.021)	0.203 (0.024)	0.203 (0.024)	0.204 (0.022)	0.198 (0.023)	0.202 (0.024)	35
North and east livestock and cereals (Z7)	0.183 (0.014)	0.18 (0.017)	0.181 (0.016)	0.179 (0.016)	0.18 (0.016)	0.179 (0.014)	13
Southeast cereals, livestock, forestry and fauna (Z9)	0.213 (0.220)	0.219 (0.020)	0.219 (0.020)	0.221 (0.018)	0.212 (0.022)	0.219 (0.018)	21
Overall	0.225 (0.048)	0.230 (0.047)	0.229 (0.047)	0.231 (0.047)	0.225 (0.047)	0.229 (0.047)	130

Note: The sum over the livelihood zones does not equal the country‐level sample size. Only livelihood zones with sample sizes greater than five are represented in the table.

**Figure 6:**
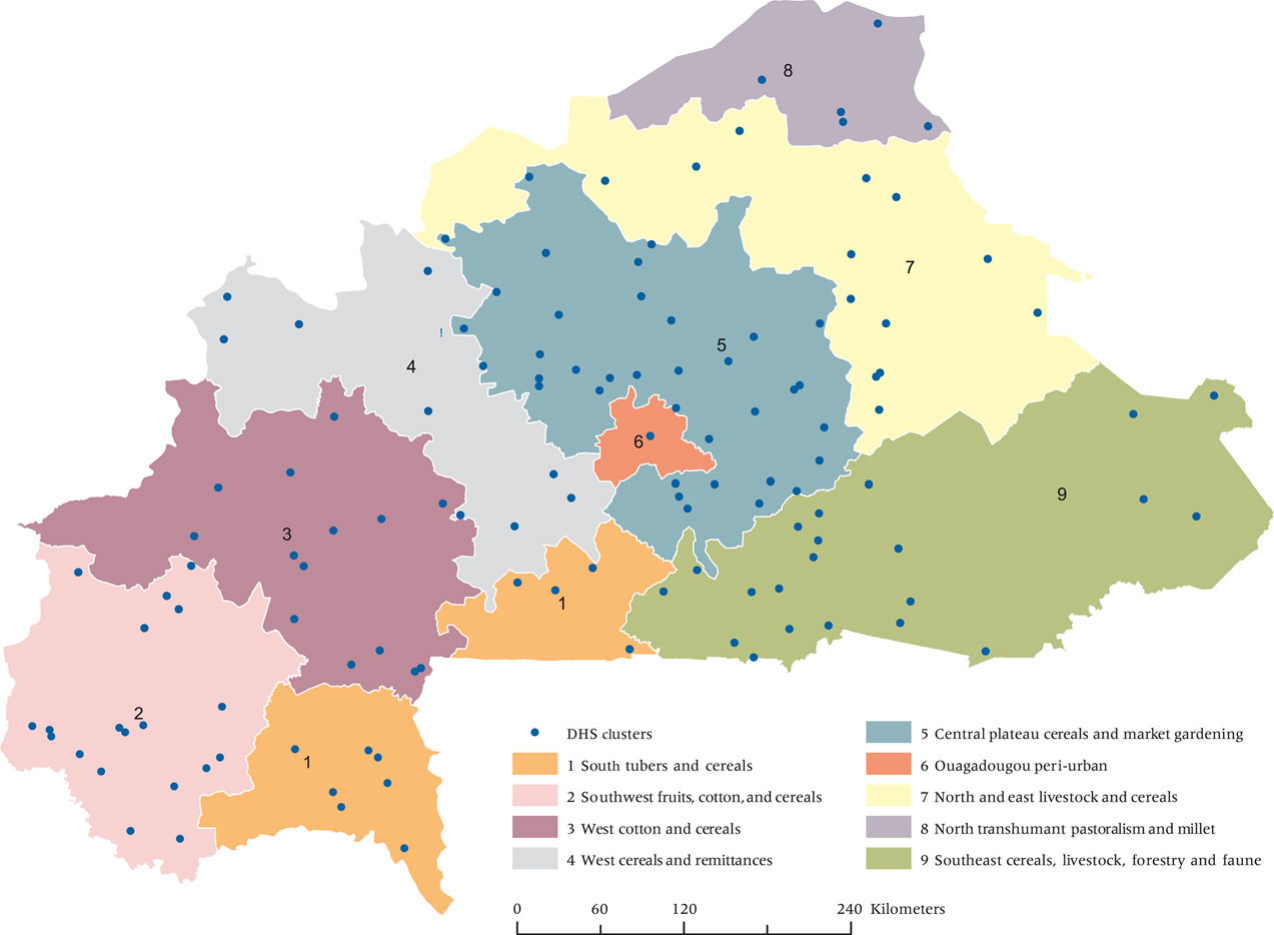
Livelihood Zones and DHS public released clusters used in analysis—Burkina Faso

Figure 6 presents the livelihood zone map and DHS locations for Burkina Faso which guides the next portion of the analysis. We account for differences in landscapes using the livelihood zones, and then compare the values across livelihoods. For livelihood zones 2, 3, and 4, we reject the null hypothesis for hypothesis 1 (p = 0.02, 0.02, and 0.06, respectively) and we fail to reject the null hypothesis for the remaining zones. In the case of hypothesis 2, we reject the null for zones 1, 2, and 4 (p = 0.07, 0.05, and 0.07 respectively). We note that many of the livelihood zones in Burkina Faso have relatively small sample sizes, possibly affecting the statistical power.

### Tajikistan

In the case of Tajikistan, our results suggest that there is a statistical difference in the differences calculated when comparing the DHS recommended approach to our alternative approach for hypothesis 1 but not for hypothesis 2 (p <0.01 and 0.96, respectively). When comparing the values across the different types of locations and buffers and while accounting for livelihood zones, a statistically significant difference in the values is revealed for hypothesis 1 and for hypothesis 2.

**Figure 7:**
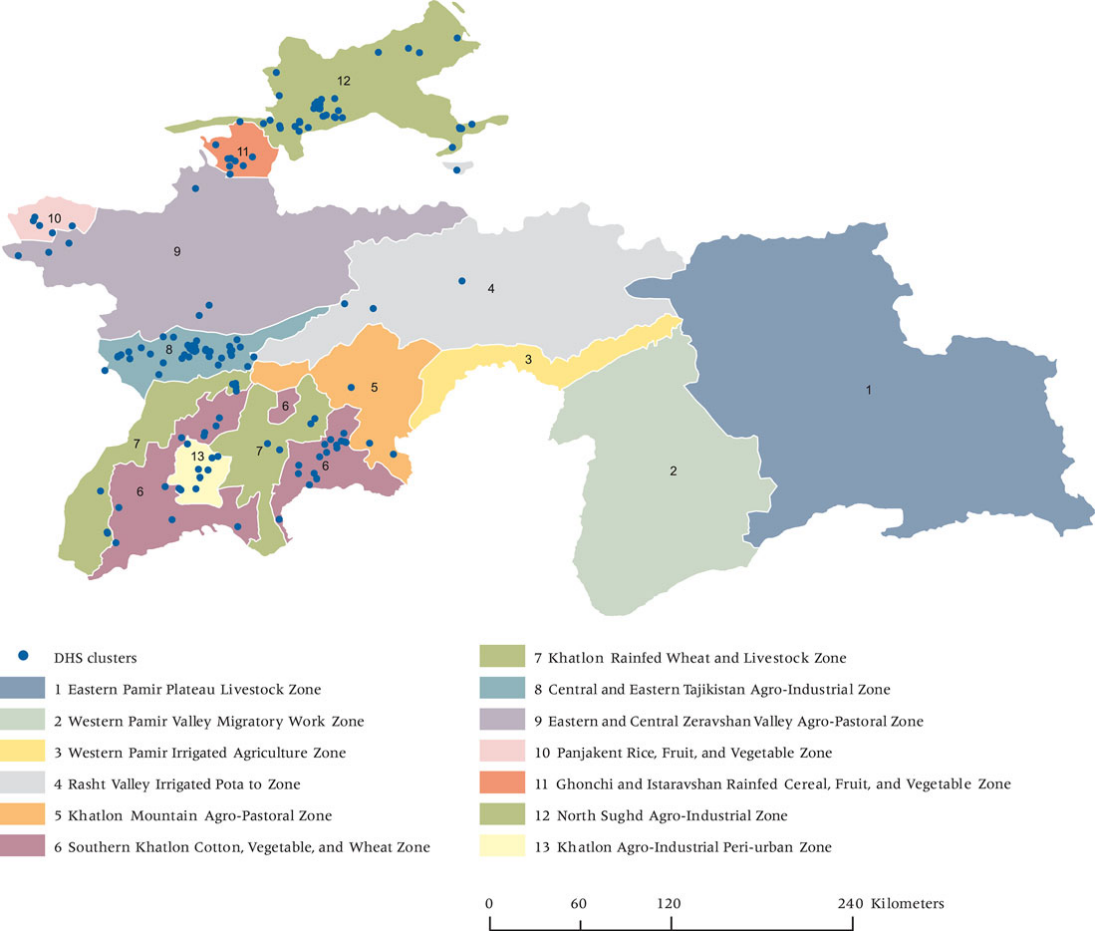
Livelihood Zones and DHS public released clusters used in analysis—Tajikistan

The median NDVI values of the settlements using a 5 km buffer is closer to the true location than when using the public DHS geocodes with a 10 km buffer for some livelihood zones (see Figure 7). This result is not equivalent across all zones, however. In the cases of zones 6, 7, 8, and 12, the differences in the true and estimated NDVI values are statistically smaller (p = 0.02, 0.06, 0.02, and 0.08, respectively) when using settlements than when using the DHS geocodes. For hypothesis 2, using the smaller buffers, the settlement approach used in zone 12 produces results that are closer to those of the true locations as compared to the DHS approach (p = 0.04). This result highlights the “place”‐specific nature of environmental context as measured by NDVI. Possible explanations include different activity spaces under different land‐use contexts or given different levels of wealth and development as captured by livelihood zone. Table 2 provides the mean values, standard deviations, and sample sizes overall and according to each livelihood zone.

**Table 2:** Means and standard deviations of NDVI calculated for the settlement location, for the DHS cluster location, and for the true location—Tajikistan

	Settlement		Public		True Points		
Livelihood Zone	2 km	5 km	5 km	10 km	2 km	5 km	N
Southern Khatlon cotton, vegetables and wheat (Z6)	0.272 (0.062)	0.284 (0.068)	0.287 (0.076)	0.298 (0.084)	0.276 (0.133)	0.284 (0.061)	26
Khatlon rainfed wheat and livestock (Z7)	0.256 (0.099)	0.273 (0.121)	0.280 (0.124)	0.272 (0.122)	0.272 (0.119)	0.281 (0.122)	11
Central and eastern agro‐industrial (Z8)	0.130 (0.084)	0.126 (0.083)	0.117 (0.080)	0.121 (0.079)	0.115 (0.099)	0.116 (0.081)	59
Eastern and central agro‐pastoral (Z9)	0.072 (0.152)	0.043 (0.167)	0.037 (0.171)	0.039 (0.173)	0.093 (0.039)	0.046 (.167)	6
Panjakent rice, fruit and vegetable (Z10)	0.022 (0.083)	0.014 (0.083)	0.016 (0.069)	0.013 (0.059)	0.045 (0.111)	0.014 (0.072)	5
Rainfed cereal, fruit and vegetable (Z11)	0.359 (0.055)	0.343 (0.027)	0.342 (0.042)	0.332 (0.032)	0.358 (0.100)	0.347 (0.055)	9
North Sughd agro‐industrial (Z12)	0.139 (0.070)	0.120 (0.060)	0.121 (0.062)	0.106 (0.061)	0.134 (0.132)	0.123 (0.079)	38
Khatlon agro‐industrial peri‐urban (Z13)	0.194 (0.022)	0.228 (0.033)	0.236 (0.051)	0.270 (0.052)	0.225 (0.115)	0.230 (0.050)	9
Overall	0.169 (0.113)	0.165 (0.118)	0.164 (0.121)	0.164 (0.124)	0.164 (0.118)	0.163 (0.120)	171

Note: The sum over the livelihood zones does not equal the country‐level sample size. Only livelihood zones with sample sizes greater than five are represented in the table.

### Kenya

Our final country is Kenya. At the country level, the results for Kenya indicate that the settlement approach produces estimates that are closer to the true values than the DHS approach when using the largest buffers (5 and 10 km) (p = 0.01). However, when using buffers of 2 km for the settlement and true points and a 5 km buffer for the DHS geocodes (hypothesis 2), the differences in the settlement estimates and true values produce results that are not statistically different from those derived from the displaced DHS locations (p = 0.96).

**Figure 8:**
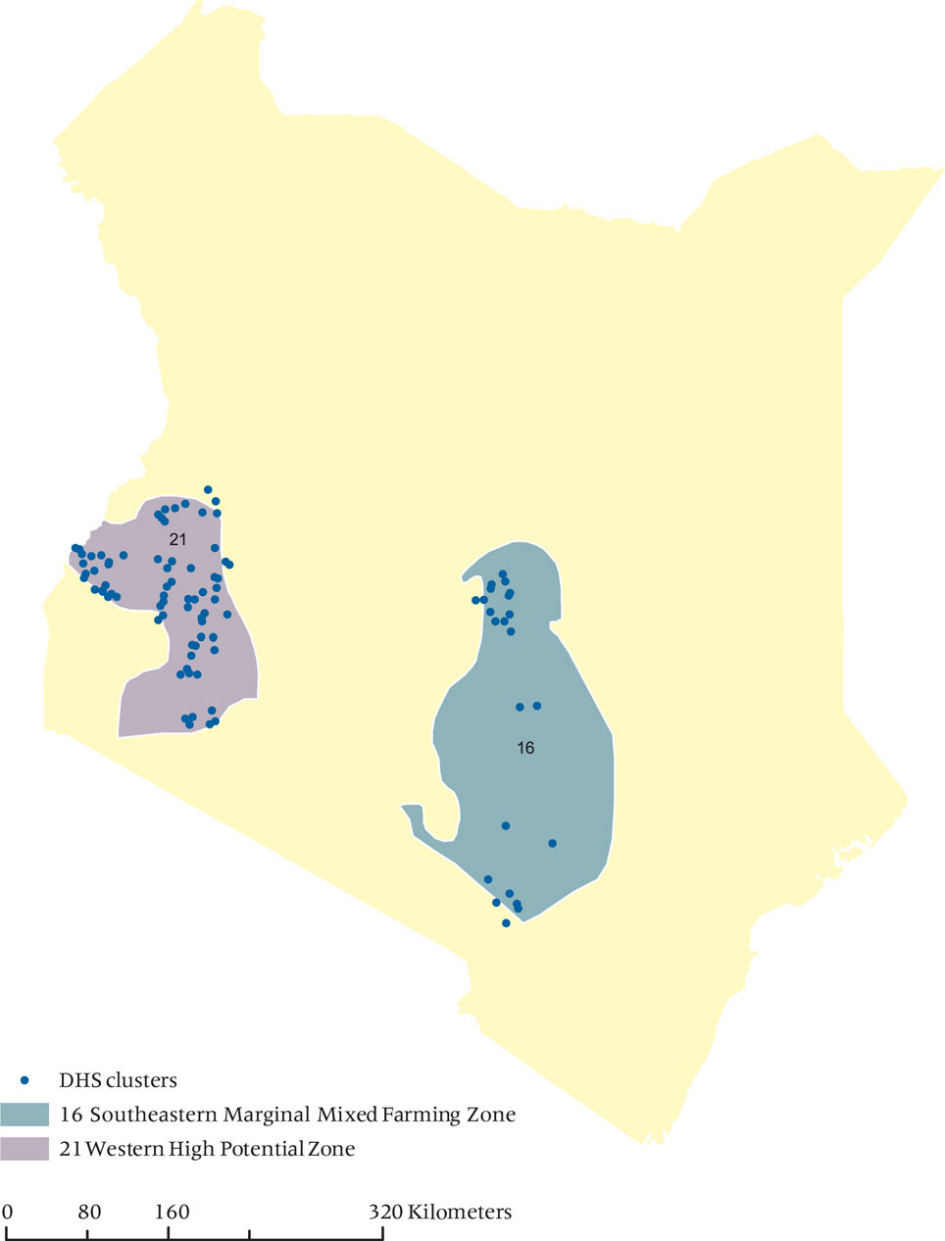
Livelihood Zones and DHS public released clusters used in analysis—Kenya

The Kenyan sample size in this study is relatively small and dividing the analysis into livelihood zones is therefore challenging. We only have two zones with adequate samples to compare values (see Figure 8)—the high potential farming zone where cash cropping is common and farms are often larger than in other areas of the country, and the marginal zone where farmers are more likely to be involved in subsistence farming and where rainfall is relatively less suited to ideal growing conditions. The results indicate that in Zone 16 (the marginal zone), the use of the settlement approach produces a vegetation value that is closer to the vegetation value of the true location when using the larger buffer (hypothesis 1, p = 0.02). When using the smaller buffer (hypothesis 2, p = 0.85), the results indicate that there is no significant difference in the results produced from the two approaches. The reason for the differences to appear at these scales and for these livelihood zones may be because subsistence farming under poorer conditions requires different areas of land as compared to farming under better conditions, i.e., larger activity space is needed to describe the land use of communities in this part of Kenya. The differences between agricultural‐ and non‐agricultural based vegetation cannot be identified when using smaller buffers.

Table 3 provides the mean values, standard deviations, and sample sizes overall and according to each livelihood zone. While many other livelihood zones were represented in our analysis, the sample sizes were often only 1 or 2 points per zone.

**Table 3:** Means and standard deviations of NDVI calculated for the settlement location, for the DHS cluster location, and for the true location—Kenya

	Settlement		Public Points		True Points		
Livelihood Zone	2 km	5 km	5 km	10 km	2 km	5 km	N
Southeastern marginal mixed farming (Z16)	0.529 (0.152)	0.530 (0.142)	0.537 (0.148)	0.520 (0.122)	0.538 (0.155)	0.535 (0.147)	10
Western high potential (Z21)	0.602 (0.096)	0.614 (0.104)	0.616 (0.107)	0.621 (0.102)	0.614 (0.103)	0.614 (0.102)	65
Overall	0.590 (0.111)	0.601 (0.115)	0.604 (0.119)	0.603 (0.112)	0.603 (0.118)	0.603 (0.116)	94

Note: The sum over the livelihood zones does not equal the country‐level sample size. Only livelihood zones with sample sizes greater than five are represented in the table.

To summarize the country‐level results, when using a 10 km buffer for the DHS locations and a 5 km buffer for the settlements and true values, there is a significant difference (improvement with the settlement approach) for each of the three countries (hypothesis 1). Alternatively, when using the smaller buffers of 5 km (around the DHS cluster) and 2 km (around the true and settlement locations), there is a significant improvement (i.e., the settlement‐based value is closer to the true value) for Burkina Faso (hypothesis 2). These outcomes reflect the heterogeneity in the landscape and the level of development of the country which together affect the activity space in terms of agriculture and land use. Table 4 summarizes the country‐level permutation test results for each of the three countries.

**Table 4:** Summary of country‐level median ratios and corresponding confidence intervals for hypotheses 1 and 2

	Burkina Faso		Kenya		Tajikistan	
	Median of Ratio; 95% CI^a^	p‐value	Median of Ratio; 95% CI^a^	p‐value	Median of Ratio; 95% CI^a^	p‐value
Hypothesis 1	0.82	<0.01	0.78	0.01	0.73	<0.01
$H0\;\;:\mathrm{MSE}\left(x_{1}^{5}-x_{T}^{5}\right)-\mathrm{MSE}\left(x_{0}^{10}-x_{T}^{5}\right)=0$ vs. $\mathrm{HA}\;\;:\mathrm{MSE}\left(x_{1}^{5}-x_{T}^{5}\right)-\mathrm{MSE}\left(x_{0}^{10}-x_{T}^{5}\right)<0$	(0.65, 0.94)		(0.62, 0.91)		(0.63, 0.85)	
Hypothesis 2	0.82	0.04	1.21	0.96	1.26	0.97
$H0\;\;:\mathrm{MSE}\left(x_{1}^{2}-x_{T}^{2}\right)-\mathrm{MSE}\left(x_{0}^{5}-x_{T}^{2}\right)=0$ vs. $\mathrm{HA}\;\;:\mathrm{MSE}\left(x_{1}^{2}-x_{T}^{2}\right)-\mathrm{MSE}\left(x_{0}^{5}-x_{T}^{2}\right)<0$	(0.69, 0.99)		(0.99, 1.65)		(1.01, 1.50)	

a note: bootstrapped confidence intervals

### Discussion

Our analysis provides quantitative information useful for any spatial investigation of survey data that uses an anonymized approach to spatial information by shifting the publicly available spatial coordinates. It also provides a framework for conducting spatially relevant analyses of various population outcomes and the environment. In the context of climate change, concerns over clean water and adequate environmental resources abound, yet population data rarely contain the environmental information necessary to investigate the most pressing population‐environment issues. The results here provide a geographically‐grounded approach that exploits the fact that people who live near each other likely do things more similarly to each other. Our results also highlight the place‐specific nature of land use or the relevant activity space. The analyst, however, must carefully consider each situation/community/area under study to select the “best” spatial approach.

Our case studies—Burkina Faso, Kenya, and Tajikistan—describe countries with vastly different levels of economic development, agricultural strategies, population density, and topography. These differences are observable in the satellite imagery that we used to identify the settlement locations. These differences are also reflected in food insecurity outcomes, mortality levels, and many other indicators. Our results, however, highlight the importance of these contexts when an analyst considers how to respond to the displacement of survey geocodes to maintain confidentiality of participants. Instead of using a 10 km buffer around a point with the assumption that the relevant values of the true location will be included in the buffer, our results demonstrate that it is potentially justifiable to locate a nearby settlement and calculate measures using a more precise activity buffer that corresponds to that location.

Our settlement identification strategy relied on manual interpretation of satellite imagery, which may be too time consuming for a multi‐country analysis or an analysis covering multiple time steps (e.g., multiple years of DHS data for a given country). To minimize the resources devoted to settlement identification, researchers may consider using existing gridded population data such as the Global Human Settlement Layer [GHSL] (Pesaresi et al. 2015), Global Urban Footprint (Esch et al. 2017; Esch et al. 2011), the population census‐based Gridded Population of the World v.4 (CIESIN 2017), the population census and ancillary data‐based WorldPop (www.worldpop.org.uk) or LandScan (http://web.ornl.gov/sci/landscan/) to create synthetic “settlements” as inputs to their spatial analysis. It is imperative, however, that researchers understand the analytical scale of these gridded datasets. For instance, Gridded Population of the World v.4 does not use any specific information about settlements beside census boundaries, which may be too coarse. Similarly, older versions of WorldPop were based on buffers around settlement points, which do not significantly improve on our own approach. Future versions of WorldPop, as well as LandScan, may rely on high resolution imagery to define settlement boundaries in ways that could automate and improve our approach. Regardless of how settlements are identified, we believe that this research shows that scholars will still need to determine the proper environmental context for their studies.

Naturally, this approach is only conceptually justifiable when the measure of interest reflects some type of interaction between the natural environment and the people who live in that space. For exogenous environmental variables like rainfall and temperature, the approach described here would typically not be useful. However, investigations of water quality, agricultural production, environmental degradation, and many other related topics could benefit from the approach described here. Another consideration is that, in some cases, settlements might form near particular environments (e.g., bodies of water) and, thus, vegetation differences as observed here might reflect behavioral responses to environmental conditions. In other words, the local environment might drive settlement formation rather than humans causing changes in the local environment. Regardless of the causal relationship between vegetation and human settlement patterns, considering settlement conditions seems to provide important insight in a number of the cases investigated in this research.

In practice, we recommend that researchers consider the heterogeneity of the landscape immediately around villages versus more remote landscape. In some places, as in the case of Burkina Faso, these landscapes may be quite different. In contrast, more intensive agricultural development, as exhibited in some of areas of Tajikistan, can lead to more homogenous landscapes. When the landscape is heterogeneous, we urge researchers to identify potential villages and use them for identifying environmental context. In places where the environmental context is more homogeneous there may not be a noticeable difference between using the public DHS geocodes versus using actual settlement locations.

As spatially referenced environmental and contextual data becomes increasingly accessible and relevant to health questions (in the context of climate change, for example), properly merging and matching data with different spatial and temporal resolutions is vital. Researchers who use the DHS use a variety of approaches for spatial data merging that are not necessarily ideal for capturing the environmental conditions of greatest interest. This article aimed to address the strategies commonly used and proposes a theory‐based and straightforward alternative for adding contextual environmental variables to survey data that maintains confidentiality of those surveyed.

## Notes

1
https://www.digitalglobe.com/products/digitalglobe-basemap

2 FEWS NET is funded by the USAID and is a leading provider of early warning and analysis on food insecurity – http://fews.net

3 These data are accessible through the third, seventh, and eighth authors and for use in methodological studies that look at ways to use the DHS data after the displacement confidentiality procedure.

4 We do note that under certain conditions entire villages have relocated due to flooding, for example. In future uses of this method, timing may determine the best source of remotely sensed imagery to use.
